# Spontaneous Generation of Unconventional Language and Its Link with Grammatical Performance in Chinese Adults With and Without ASD

**DOI:** 10.1007/s10803-024-06415-1

**Published:** 2024-07-05

**Authors:** Zixuan Wu, Cherry Lam, Carol K. S. To

**Affiliations:** Academic Unit of Human Communication, Learning, and Development, Faculty of Education, The University of Hong Kong, Hong Kong SAR, China

**Keywords:** Autism, Unconventional language, Grammar, Morphosyntax, Language production

## Abstract

This study investigated the generation of unconventional language in the spontaneous speech of Chinese adults with autism spectrum disorder (ASD), and how it was related to their grammatical performance, when compared to neurotypical (NT) controls. Twenty Cantonese-speaking adults with ASD and 20 NT controls completed three interview tasks in the Autism Diagnostic Observation Schedule, Second Edition (ADOS-2), and their spontaneous speech was recorded and transcribed. Utterances containing unconventional language (neologisms, idiosyncratic phrases, and pedantic language), morphosyntactic errors, mean length of utterance (MLU), and mazes were computed. The ASD group produced more neologisms, idiosyncratic phrases, and pedantic language than the NT group and their grammatical difficulties were shown in shorter MLU but not morphosyntactic errors. Mazes were more frequent in the ASD than the NT group. While the use of unconventional language increased with MLU in the NT group, it correlated positively with mazes in the ASD group. Generation of unconventional language, particularly pedantic language, in Cantonese-speaking NT adults is linked to more advanced grammar, while it appears to be a common speech characteristic among autistic speakers regardless of individual grammatical performance.

Stereotyped and idiosyncratic language use are widely identified instances of language atypicality in autism spectrum disorders (ASD). Such atypical language is also termed “unconventional language” in Luyster et al.’s (2022) framework. Luyster et al. suggested that this type of language use is categorized as non-generative and generative. Non-generative unconventional language is language produced by autistic individuals simply repeating the utterances of others (echolalia) or their own utterances (self-repetition) without accessing to their own lexical and syntactic knowledge. Generative unconventional language (GUL) is unusual language spontaneously produced by autistic speakers using their structural language knowledge (i.e., phonology, morphology, and syntax) and consists of three subtypes: neologisms (idiosyncratic words), idiosyncratic phrases, and pedantic language (Luyster et al., 2022). Apart from its generative nature, the formation of such a word or phrase is unusual. Listeners may find the meaning strange or hard to interpret or inappropriate to the context.

Although GUL is commonly recognized in standardized diagnostic assessments (e.g., Autism Diagnostic Interview-Revised [ADI-R]: Lord et al., 1994; Autism Diagnostic Observation Schedule, Second Edition [ADOS-2]: Lord et al., 2012), studies systematically investigating their nature remain scarce and variabilities in classifying these expressions were noted. Volden and Lord (1991) studied unconventional language use in ASD by examining the production of idiosyncratic language together with pedantic speech without explicit distinction of these two types. Suh et al. (2014), on the other hand, discussed unusual language expressions among autistic individuals by drawing differences among pedantic language, neologism, and scripted language (a kind of non-generative echolalia), explicitly considering pedantic language as a separate category. Such a differentiation in analysis is deemed more informative because pedantic language is more complex and represents a more formal percept than other types of unconventional languages and occur more often in written discourse (Ghaziuddin & Gerstein, 1996). The diverse classification systems may introduce challenges when examining the links between the use of these language forms and other autistic phenotypes.

Luyster et al. (2022) put forward a new framework of GUL subtypes. According to the framework, neologisms and idiosyncratic phrases constitute idiosyncratic language: Neologism refers to the combination of phonemes or morphemes to form a novel word [e.g., “consailed” in “we consailed the King’s Cross underground” in a case study by Werth et al., (2001, p. 116); with the word “consailed” possibly indicating a kind of movement], while idiosyncratic phrases refer to the meaningful but unconventional use or combination of existing words [e.g., “go as deep as economical with it” in Volden and Lord (1991, p. 127); with the word “economical” rather than “possible” being used]. Idiosyncratic language use by autistic individuals may indicate semantic weakness, as they may fail to access the appropriate and conventional meaning (Eigsti et al., 2007; Luyster et al., 2022; Volden & Lord, 1991). The use may also demonstrate pragmatic deficits such that the speakers fail to observe the listener’s needs in a communication where shared knowledge and enjoyment are expected (Werth et al., 2001), or fail to capture the social connotations of the expression (Volden & Lord, 1991). Pedantic language, the other form of GUL, is normally characterized by rare, technical, overly accurate, complex, and formal expressions (de Villiers et al., 2007; Luyster et al., 2022), which convey a “bookish” tone of voice to the listener (Ghaziuddin & Gerstein, 1996). This is often observed in fluent autistic speakers, with an even greater prevalence in those who are diagnosed with Asperger Syndrome (de Giambattista et al., 2019; Ghaziuddin & Gerstein, 1996). This type of language may indicate the speaker’s enjoyment of words or desire for precision over efficiency during communication (Luyster et al., 2022). It was also discussed by some researchers in terms of the pragmatic impairment of providing excessive information relative to the conversation needs (Ghaziuddin & Gerstein, 1996). The frequent use of pedantic language may make listeners feel distant, and presents challenges for autistic individuals in developing interpersonal relationships.

Given its generative nature, production of GUL may reflect the structural language abilities of the speakers. Attempts have been made to explore how the use of GUL is related to or can be explained by structural language performance, especially grammar. Two proposals have been put forward to account for the possible relationship, based on the data of English-speaking autistic individuals. One is the developmental asynchrony hypothesis proposed by Volden and Lord (1991). They viewed GUL as semantic errors and stated that increased grammatical complexity of the utterances increases the possibility of GUL and also morphosyntactic errors. Their study, however, considers pedantic language a type of idiosyncratic language and was not coded separately. Pedantic language is semantically complex, its correlation with grammatical complexity would be stronger than other GUL subtypes. Without a subtype analysis, the pattern of unconventional language and its link with grammar is still unclear.

The other explanation is that the use of GUL among autistic individuals may be associated with their morphosyntactic difficulty (Eigsti et al., 2007). Eigsti and colleagues examined the morphosyntactic ability in autistic children. The use of “jargons”, which were defined as intelligible but uninterpretable expressions, was coded and its correlation with the morphosyntactic ability was explored. In relation to the current study, “jargons” covered mainly neologisms and possibly some idiosyncratic phrases, and pedantic language was not counted. The authors claimed that the more frequent use of jargon by the autistic participants, the less advanced the grammatical skills, as measured by the Index of Productive Syntax (IPSyn; Scarborough, 1990). However, the claim was weakened by the non-significant correlation between the use of jargon and IPSyn scores. In addition, the IPSyn measure considered the frequency of specific grammatical structures irrespective of their well-formedness. Even a measure of grammatical errors was included in Eigsti et al. it mainly concerned about omission, and their autistic children performed similarly as the younger controls. A more systematic analysis of grammatical errors may provide a valid measure to test the hypothesis of GUL being an indicator associated with grammatical deficits.

## The Present Study

Previous findings regarding the nature of GUL used by autistic individuals and the relationship with grammar appear to be equivocal. It remains to be examined whether there is a positive relationship between GUL and grammar in autistic individuals and how the relationship is different with different types of GUL. The present study investigated the use of GUL by Cantonese-speaking autistic adults and its association with grammar, by adopting a more fine-grained classification system of GUL and the grammatical measures representing both complexity and error frequency. Luyster et al.’s (2022) framework of GUL subcategories was adopted. Grammatical skills were investigated in terms of a global measure of mean length of utterances (MLU) and also morphosyntactic errors. Linguistic mazes were also coded. The current study focused on the performance of adults whose language abilities are relatively stable (Girolamo & Rice, 2022), because previous studies have demonstrated improvements in the use of GUL and grammatical ability across the age span from preschool years to adolescence (Fecteau et al., 2003; Starr et al., 2003; Volden & Lord, 1991), leading to challenges to tease apart developmental changes and ASD-related errors.

Clinically, the use of unusual languages is commonly observed in autistic individuals who speak Cantonese and can be easily noticed by native typical speakers. In particular, pedantic language may be the most noticeable GUL subtype. This is because Cantonese is basically a spoken variety and the written form follows Modern Standard Chinese (MSC) which is slightly different in syntax and to a larger extent the vocabulary (To et al., 2012). About one third of Cantonese words are different from the corresponding form in MSC (Li et al., 1995). For example, *gwai6tung2* (“drawer”) is only used orally, whereas its written MSC form is *cau1tai3*. Generally speaking, the oral form is used in informal situations, while the written form (MSC expressions) corresponds to the formal form. This draws a clearer formality distinction in registers when compared to English where there is a higher overlap between oral and written forms. Studying GUL in the Cantonese ASD group, in particular pedantic language that previous studies on English left unexplored (Eigsti et al., 2007; Volden & Lord, 1991), may provide a more comprehensive understanding of the unconventional language pattern used among autistic speakers.

Morphosyntactic performance of English-speaking autistic individuals displays large heterogeneity, with some showing intact ability while some showed specific expressive morphosyntactic errors such as tense markers (Girolamo & Rice, 2022; Modyanova et al., 2017; Roberts et al., 2004; Tager-Flusberg, 2006), articles, auxiliaries, and copula (Bartolucci et al., 1980; Girolamo & Rice, 2022). The results of the previous studies using the global language measure of MLU as a proxy of structural language skills may be confounded by the potential grammatical errors used by the autistic speakers.

Compared to English, Chinese’s inflectional morphology is much more reduced. Errors related to tense and articles discussed in the English literature are absent in Chinese. Studies on Chinese-speaking autistic individuals have reported specific grammatical errors in aspect markers using controlled sentence elicitation tasks (Chen et al., 2022, 2023; Zhou et al., 2015). However, in spontaneous discourse, aspect marker is not obligatory, which means its errors can be circumvented by using lexical alternatives to convey aspectual information (Stokes & Fletcher, 2003). Grammatical errors may be relatively less prominent issue among Cantonese-speaking autistic individuals when compared to their English-speaking peers. Cantonese may therefore provide a fruitful and fair testing ground to explore the relationship between grammatical skills (as measured in terms of MLU) and GUL produced by autistic speakers.

Based on the previous studies on English and our clinical observation, we hypothesized that, Cantonese-speaking autistic adults would produce more GUL than their age-matched NT controls. Among the GUL subtypes, we expected to observe a large proportion of pedantic language in the ASD group, given the relatively clear formality distinction in Cantonese. We also anticipated a lower grammatical performance in the ASD group (i.e., shorter MLU) and based on Volden and Lord (1991), we expected the frequency of GUL, specifically pedantic language, would increase with utterance complexity as measured in terms of MLU in the autistic group.

## Methods

### Participants

Twenty adults with ASD (2 females, aged 20–34, *M* = 27.50, *SD* = 3.80) participated in the present study. They were recruited from the non-governmental organizations (NGOs) in Hong Kong that provide vocational training to adults with high-functioning autism before they joined their workplace. All had received a formal diagnosis of Autism, Pervasive Developmental Disorder-Not Otherwise Specified (PDD-NOS) or Asperger Syndrome from a clinical psychologist or a pediatrician during their childhood. To confirm the diagnostic status of ASD at the time of the testing, the ADOS-2 Module 4 (Lord et al., 2012) was administered to the participants by a research-qualified personnel. Seventeen participants were classified as having “autism” and three fell in the range of “autism spectrum”. All the participants were native Cantonese speakers who had received compulsory education in local mainstream schools at least up to Form 5 level. The nonverbal IQ of the autistic participants was assessed with the Test of Nonverbal Intelligence (TONI-4; Brown et al., 2010). The participants displayed average to above average level with the mean index score of 104.33 (*SD* = 12.10).

Twenty Cantonese-speaking NT adults matched on age (*t* = − 0.369, *p* = 0.357), sex, and education with the ASD group were recruited using convenience sampling as the control group (2 females, aged 22–36, *M* = 27.05, *SD* = 3.90). The Autism Spectrum Quotient (AQ; Baron-Cohen et al., 2001) was used to confirm that the scores for autistic traits in the NT group fell within the normal range. Written informed consents were obtained prior to the study.

### Procedure

Speech samples were collected from the ASD group during the interviews using ADOS-2 module 4 (Lord et al., 2012). This module is a standardized assessment of autism designed for verbally fluent older adolescents and adults. Participants were interviewed individually by a research-qualified ADOS-2 assessor. Data used for analysis comprised speech samples collected during three interviewing tasks involving narrative or conversational elements: (a) telling a standard story from a wordless picture book, (b) describing a cartoon picture illustrating tourist spots and scenery, and (c) talking about current work or school issues in a spontaneous conversation guided by default questions. Both narrative and conversational tasks were included because the genres are more natural, and would provide more opportunities for the use of GUL than well-controlled sentence elicitation tasks. Speech samples from the NT group were also elicited using the above three tasks. All the interviewing procedures were videotaped for subsequent analysis.

### Analysis

#### Transcription and Coding

The videotaped speech samples were transcribed verbatim. Partially unintelligible utterances due to unclear speech or recording were identified. Transcribers attempted to resolved the unclarity by supplementing their interpretation based on the contextual cues in the videos. Utterances were mainly segmented based on intonation, with the reliance on syntax to a lesser extent (To et al., 2010) given the important role of prosody in marking Chinese sentence boundaries (Chao et al., 1968). Following the frameworks developed by Luyster et al. (2022) and the definition in Volden and Lord (1991), GUL coded in this study included neologisms, idiosyncratic phrases, and pedantic language. Table 1 summarizes the examples of the three GUL types extracted from the present study. *Neologisms* were defined as words or phrases that were nonexistent in the lexicon of Cantonese. *Idiosyncratic phrases* were defined as the unusual selection or combination of conventional Cantonese words or phrases. They also included unconventionally metaphorical forms.

**Table 1:**
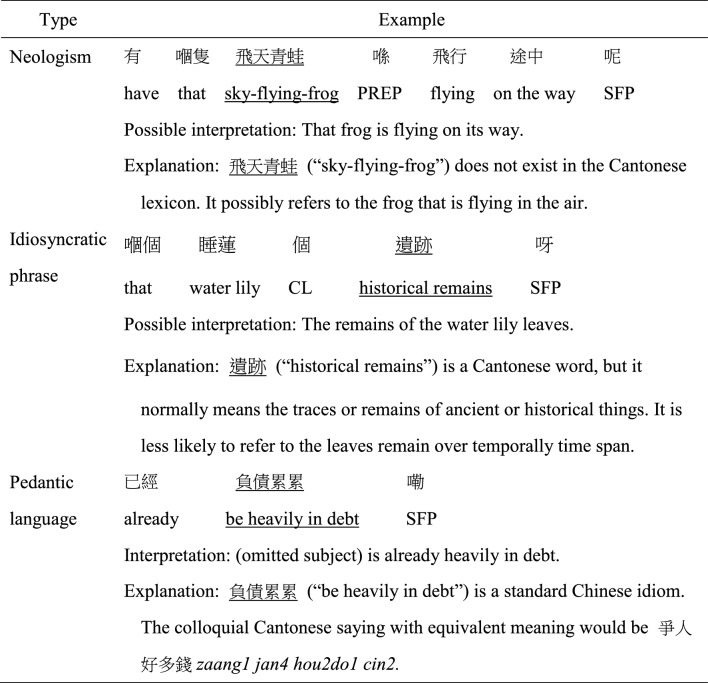
Examples of generative unconventional language subtypes

Underlined words or phrases represent the occurrence of generative unconventional language

*PERP* preposition; *SFP* sentence-final particle; *CL* classifier

*Pedantic language* was defined as overly complex and formal expressions in a casual context, conveying an impression of “bookish” to the listener. The judgement of pedantic language was a subjective call but typical native speakers would be sensitive to them. Operationally, pedantic language included the MSC expressions (i.e., the formal form) for which the message is typically expressed in a way that is different from the colloquial forms in casual speech. The difference can be realized in vocabulary (e.g., using the written vocabulary *cau1tai3* instead of the oral form *gwai6tung2* for “drawer”) or syntax (e.g., the existential form of MSC, *zau2ceot1jat1zek3maau1lai4* “(somewhere) comes a cat” is not often used in spoken Cantonese). Traditional Chinese four-character idioms were also included in pedantic language, as they normally occur in formal registers and written discourse.

MSC and Cantonese share many similarities in term of grammatical rules. However, certain syntactic structures may be more frequently used in either MSC or Cantonese (e.g., more frequent use of *Ba* sentence in MSC), but those structures are considered well-formed in both MSC and Cantonese. A morphosyntactic error was coded when the structural elements in an utterance violated the grammatical rules of colloquial Cantonese. Twelve subtypes of morphosyntactic error were coded: errors with classifiers, possessive particle *ge3*, objects, other noun phrase errors, pronouns, verbs, aspect markers, verbal particles, prepositions, conjunctions, word order, and other unclassified errors not belonging to the above categories, as exemplified in Table 2. As a “pro-drop” language, Cantonese and MSC allow the omission of subjects if the omitted constituent is the topic having been provided previously or can be clearly inferred through context (Matthews & Yip, 2011; To et al., 2013). Therefore, appropriate omission of subject, in form of ellipsis which marked discourse-level cohesion, were not regarded as an error. However, omission error in object positions was more frequently coded as an error when the omission was ungrammatical, sounded unnatural to the coders or the antecedents cannot be resolved.

**Table 2:**
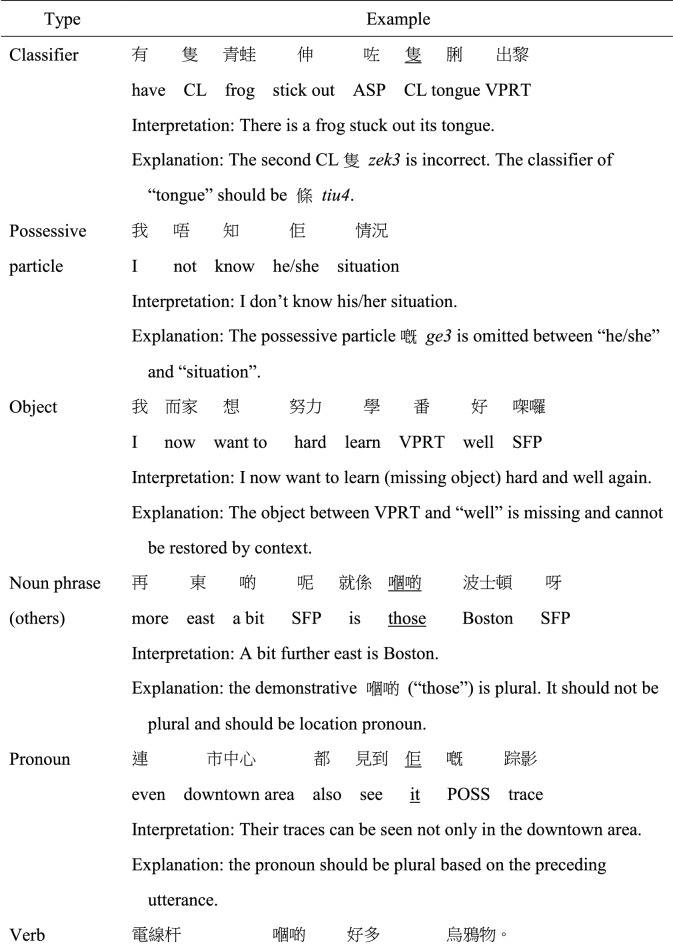

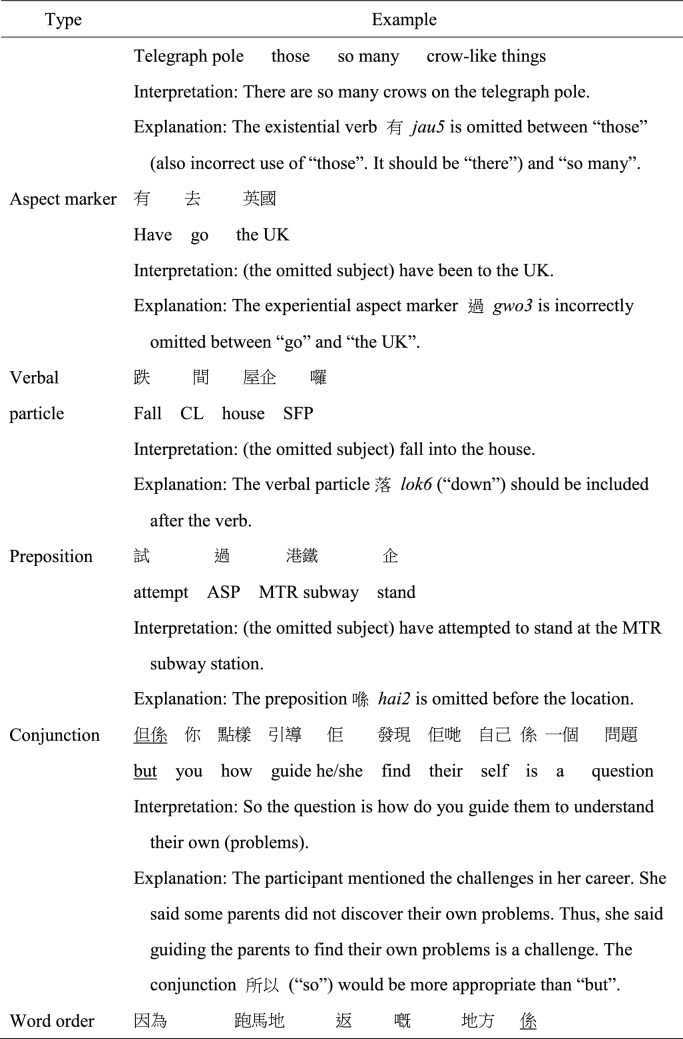

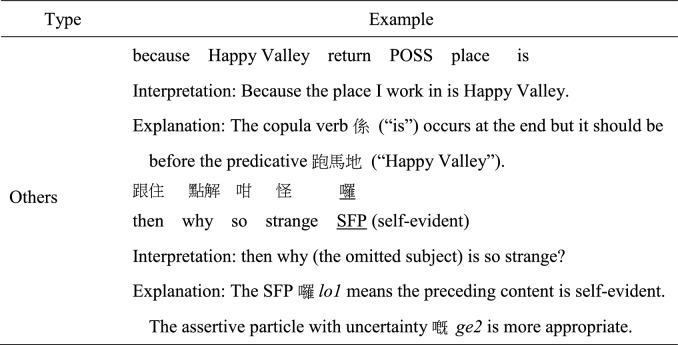
Examples of morphosyntactic error subtypes

*CL* classifier; *ASP* aspect marker; *VPRT* verbal particle; *SFP* sentence-final particle; *POSS* possessive particle

MLU was considered a proxy for the grammatical complexity of the utterances generated by each participant and was computed by dividing the total number of words by the total number of utterances for each participant. Word segmentation followed Cheung et al.’s (2011) criteria. Unintelligible speech segments that could not be recovered at the transcription stage were not included in MLU calculation.

Linguistic mazes refer to disruptions or disfluencies commonly occurring in spontaneous speech and manifested in form of pauses, repetitions, revisions, and orphans (also known as abandoned utterances) (Dollaghan & Campbell, 1992). Its production may reveal difficulties in the generative process, such as utterance formation or word finding (Leadholm & Miller, 1994). Other cognitive factors, such as executive functioning (Engelhardt et al., 2013) and verbal intelligence (Engelhardt et al., 2017, 2019) are also found to be related to the production of mazes. The present study mainly focused on three types of mazes: repetitions, revisions, and orphans; pauses were not analyzed. GUL and morphosyntactic errors and mazes were identified and coded by undergraduates of the Bachelor’s degree in Speech and Hearing who were all native Cantonese speakers. MLU was calculated in Computerized Language Analysis (CLAN, MacWhinney, 2000).

#### Reliability

All the samples were double-coded independently by two coders. Both coders had taken undergraduate courses on Cantonese linguistics and are native Cantonese speakers. They coded the transcribed data and were unaware of participants’ group membership. Reliability was calculated by the total number of point-by-point agreement divided by the sum of match and mismatch numbers (Schroeder et al., 2023). The average agreement was 90.73%. Mismatches were resolved by the last author before subsequent analysis.

#### Statistical Analysis

Given the variability in the number of utterances produced across participants, the proportions (in percentages) of utterances containing GUL (P_GUL), morphosyntactic errors (P_morphosyntactic errors), and their respective subtypes (marked with “P_” before the subtypes) were calculated. Similarly, the proportion of utterances containing linguistic mazes (P_mazes) was also measured. Q-Q plots and Shapiro–Wilk test were used to evaluate the distributional normality of the above outcome measures. Given that no measure conformed with normal distribution in both groups (*p*s < 0.05), this study used nonparametric Mann–Whitney *U* tests to investigate group differences in each measure. To reduce Type I errors made by multiple comparisons, Benjamini–Hochberg correction was conducted (Benjamini & Hochberg, 1995). The *p* values reported were the adjusted values obtained through this correction method. Also, Spearman rank-order correlations were applied separately within each group to investigate whether P_GUL had a correlation with grammar (MLU and P_morphosyntactic errors) or P_mazes. Analyses were conducted using SPSS version 28 (IBM). Graphs were made using *ggplot2* (Wickham, 2016) and *ggbeeswarm* (Clarke et al., 2023) packages in R version 4.0.2 and RStudio version 2023.03.1. Effect size (*r* value) was calculated using the formula* r* = *Z*/sqrt(*N*), where *Z* is the *z*-value, and *N* is the total sample size. The effect size was considered large if *r* reached 0.5; medium if *r* was 0.3; small if *r* was 0.1 (Cohen, 1988).

## Results

The number of utterances produced by the ASD group ranged from 136 to 450 (*M* = 225.70, *Mdn* = 202, interquartile range [IQR] = 74), while that produced by the NT group ranged from 120 to 342 (*M* = 188.75, *Mdn* = 164.5, IQR = 83).

### GUL

**Fig. 1:**
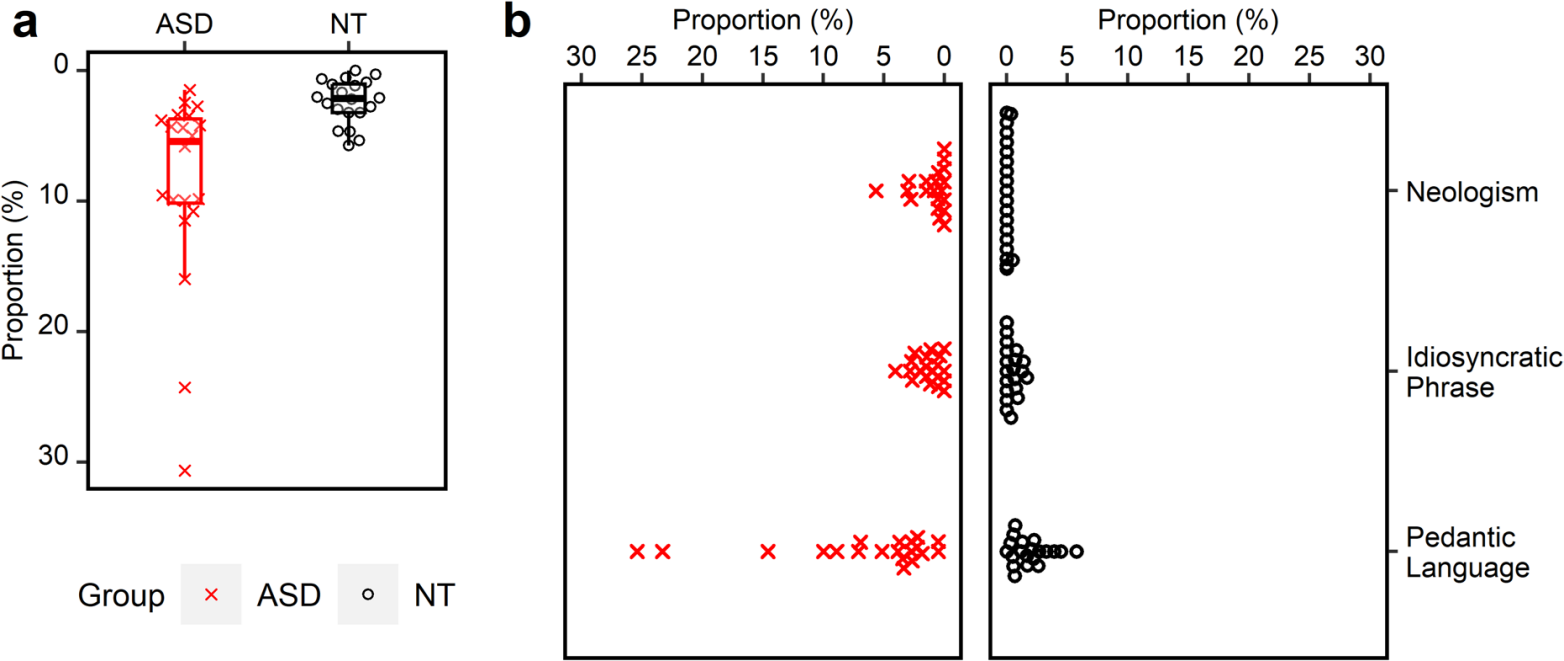
The proportions of utterances containing **a** generative unconventional language and **b** specific subtypes of generative unconventional language

Figure 1a and b display the distribution of P_ GUL and its subtypes in the ASD and the NT groups respectively. The ASD group also showed higher within-group variabilities, with the IQR greater than that in the NT group (7.02 vs. 2.29), their first quartile of the GUL production (3.55) was still higher than the third quartile of the NT group (3.24), suggesting that the GUL performance of the ASD group is beyond the individual variations of the NT group. Results of the Mann–Whitney *U* test for P_GUL showed that the proportion of the ASD group (*M* = 8.67, *Mdn* = 5.41) was significantly larger than the NT group (*M* = 2.39, *Mdn* = 2.14), with a large effect size (*U* = 344.0, *p* < 0.001, *r* = 0.616). Regarding the subtypes, the three measures, P_neologisms (ASD: *M* = 1.05, *Mdn* = 0.51, IQR = 1.49; NT: *M* = 0.04, *Mdn* = 0, IQR = 0; *U* = 320.0, *p* = 0.004, *r* = 0.590), P_idiosyncratic phrases (ASD: *M* = 1.32, *Mdn* = 1.14, IQR = 1.95; NT: *M* = 0.45, *Mdn* = 0.17, IQR = 0.77; *U* = 294.0, *p* = 0.033, *r* = 0.411), and P_pedantic language (ASD: *M* = 6.56, *Mdn* = 3.58, IQR = 6.10; NT: *M* = 1.90, *Mdn* = 1.68, IQR = 2.05; *U* = 318.0, *p* = 0.004, *r* = 0.505) were higher in the ASD group than the NT group.

### Morphosyntactic Errors and MLU

**Fig. 2:**
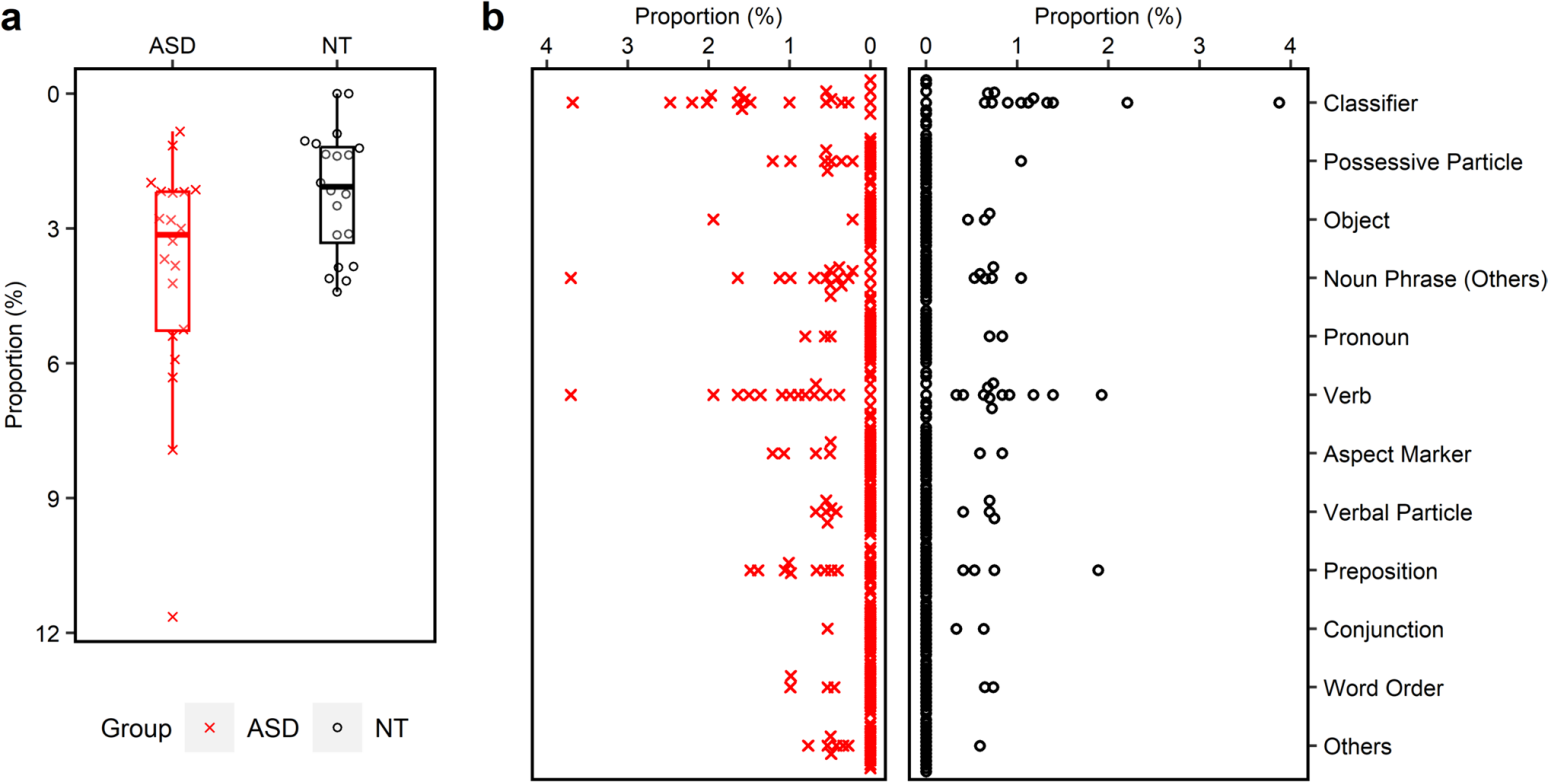
The proportions of utterances containing **a** morphosyntactic errors and **b** specific subtypes of morphosyntactic errors

Figure 2a and b illustrate the distribution of the P_morphosyntactic errors and the specific subtypes in the ASD and the NT groups respectively. Similar to the production of GUL, the within group variation of the P_morphosyntactic errors produced by the ASD group was higher than the NT group. In addition, P_morphosyntactic errors of the ASD group was generally higher than the NT group but the difference is marginally significant (ASD: *M* = 3.93, *Mdn* = 3.14, IQR = 3.17; NT: *M* = 2.20, *Mdn* = 2.07, IQR = 2.53; *U* = 282.0, *p* = 0.071, *r* = 0.351). Regarding the subtypes, no significant group difference was observed (*p*s > 0.10). The ASD group had significantly shorter MLU than the NT group, with a large effect size (ASD: *M* = 5.94, *Mdn* = 5.92, IQR = 1.52; NT: *M* = 7.51, *Mdn* = 7.68, IQR = 1.39; *U* = 46.0, *p* < 0.001,* r* = − 0.659).

### Linguistic Mazes

The P_mazes of the ASD group (*M* = 21.76, *Mdn* = 20.71, IQR = 11.77) was significantly higher than the NT group (*M* = 12.64, *Mdn* = 13.41, IQR = 7.38), with a large effect size (*U* = 330.5,* p* = 0.001, *r* = 0.558).

### Correlation Analysis

To examine whether P_GUL showed a correlation with MLU, P_morphosyntactic errors and P_maze, Spearman rank-order correlations coefficients were computed for each group (see Table 3). Results showed that among the ASD individuals, P_GUL was positively correlated with the P_maze (*p* = 0.025) but no significant correlations were found between P_GUL and MLU as well as between P_GUL and P_morphosyntactic errors. In the NT group, a positive correlation was only found between the P_GUL and MLU (*p* = 0.029), suggesting that NT controls with longer MLU were more likely to show higher P_GUL than those with shorter MLU. However, their GUL production was not related to their maze production or the morphosyntactic errors.

**Table 3:** Correlations between generative unconventional language and MLU, morphosyntactic errors, and mazes

	ASD group	NT group
MLU	0.108	0.489*
Morphosyntactic errors	0.247	− 0.078
Mazes	0.499*	− 0.057

Reported values are Spearman rank-order correlation coefficients *r*_*s*_

*p < 0.05

Correlation coefficients were computed separately for neologisms, idiosyncratic phrases, and pedantic language. Results showed that for idiosyncratic language, both groups showed non-significant correlation between the P_idiosyncratic language and the measures of MLU, P_morphosyntactic errors, or P_maze. For pedantic language, positive correlations were observed between P_pedantic language and P_maze among the ASD individuals (*r*_*s*_ = 0.565, *p* = 0.009) and between P_pedantic language and MLU in the NT group (*r*_*s*_ = 0.475, *p* = 0.034). Correlations between P_morphosyntactic errors and pedantic language were not significant in both groups.

## Discussion

This study investigated the GUL production and its relationship with the grammatical performance in Cantonese-speaking adults with and without ASD. Using a narrative task, a picture description task, and a conversational task, the study showed that the Cantonese-speaking adults with ASD were more prone to produce unconventional language than the NT controls. At the same time, the speech of the ASD group was characterized by shorter MLU and more utterances containing mazes. In the ASD group, the P_GUL did not change with MLU and P_morphological errors. Instead, it increased with the P_mazes. However, in the NT group, GUL was positively correlated with MLU.

### GUL

The significantly more frequent occurrence of GUL in the ASD group than the NT group confirmed that it is one of the characterizing features of autistic speech (de Giambattista et al., 2019; Fecteau et al., 2003; Ghaziuddin & Gerstein, 1996; Starr et al., 2003; Suh et al., 2014). Among the GUL subtypes, pedantic language had the largest proportion in both the ASD and NT groups, followed by idiosyncratic phrases, and then neologisms. This was in line with the prediction, as the marked differences between informal and formal forms in Cantonese make pedantic language very easy to be detected. Pedantic language was observed in all 20 ASD participants and 19 of the 20 NT participants. The large proportion of the NT participants producing pedantic language was not expected. It is speculated that the discourse genre may play a role. Narrative is a genre falling between the formal and informal register continuum. Speakers may be prone to produce slightly more formal expressions in the narrative task than causal dialogue in order to add more color to the story being narrated. The ASD group, however, still produced significantly higher proportion of utterances that included pedantic language than the NT controls. An explanation may be that the autistic individuals showed a reduced awareness of register variations and knowledge of vocabulary. Typically, mature speakers should be able to adapt to a situation’s formality relatively instantaneously—they weigh the alternative lexicons and select the word that best suits the current situation and intention. Such situation-appropriate language use is a gradual process that develops through diverse social interactions from childhood to adulthood (Ravid & Tolchinsky, 2002). For autistic individuals with reduced exposure to social communication, they may possess lower sensitivity to the register and hence atypical weighting of the candidate words, resulting in using more formal forms in daily casual communication.

However, autistic individuals are not completely unaware of the difference in registers. Volden and Sorenson (2009) showed that in their structured tasks, autistic children and adolescents (6 to 16 years old) were able to adjust their language, in terms of syntactic directiveness, to be nice or bossy as their language-matched typically-developing peers. For example, the autistic group was able to be less directive when being nice (e.g., “May I take a candy?”) and more direct (e.g., “Give me a candy.”) when being bossy. However, using vocabulary to mark different registers as in the current study may be subtler than using sentence types. The former requires deep vocabulary knowledge relating to its usage, and such sophisticated linguistic awareness may not be mastered by the autistic individuals.

Another possible explanation for the more frequent use of pedantic language in the ASD group may be related to their language learning style. The autistic individuals may have learned and stored the formal version of the vocabulary as the default form in their lexicon, rather than the informal oral form as the typical speakers. For young typically developing children, vocabulary is acquired mainly through conversation and social interaction in daily life. Only when they start to read, their vocabulary expands exponentially, and more formal forms are acquired through books and other written materials. Autistic individuals, especially those without language difficulties, may develop their vocabularies in a different way. They may be more ready to master vocabulary through reading texts than listening to speech. In this digital era, individuals with ASD spent long time on the internet, browsing websites of their interested topics (Banerjee, 2023). Their interests lead to repeated exposure (Rothwell et al., 2023). They are therefore more likely to memorize the information along with the language used (Boucher & Mayes, 2011). The knowledge learned can be stored long-term and retrieved more readily when compared to those learned via social interaction (Klin et al., 2007; Spiker et al., 2012). This may explain why they tend to retrieve many formal and technical terms in MSC and used in their speech. Their performance is like the “code-mixing” behaviors in bilingual speakers (Bruche-Schulz, 1997). If the autistic speakers have to use informal language, they may need to suppress the activation of those formal forms through inhibitory control which may be a weakness of the autistic individuals (e.g., Kana et al., 2007; Mosconi et al., 2009). As a result, using casual language may require more effort than formal ones.

Neologisms were also more likely to occur in autistic adults than their NT controls. For NT individuals, neologisms were only observed in two participants, whose neologisms occurred in less than 0.5% of their utterances. In the ASD group, 13 out of 20 participants produced neologisms, but only two had neologisms in more than 3% of the utterances. This was generally in line with Volden and Lord’s (1991) observations that neologisms were only occasionally observed. Volden and Lord (1991) reported in their study that 29.10% of neologisms and idiosyncratic language in English showed phonological relations with the standard lexicon and revealed a non-specificity with which the word was stored (e.g., “turkey” as a possible interpretation of “turken”; Volden & Lord, 1991, p. 121). In the present study, we did a post-hoc analysis and did not find a neologism that was phonologically related with the standard lexicon. The majority of neologisms seemed to be interpretable if the chunk was analysed in terms of its component units, morphemes. For example, 飛機樹葉*fei1gei1 syu6jip6* “plane-leaf” the plane is an adjective for the head noun of leaf. Much fewer phonologically motivated neologisms in Cantonese in the present study may be because most syllables in Chinese corresponds to a morpheme, which is the basic word-formation unit in Chinese; compounding morphemes is the most productive realization of Chinese word formation, while changing phonological segments is relatively uncommon (Ceccagno & Basciano, 2007).

More idiosyncratic phrases were also found in the ASD group than the NT group. Some phrases identified exist in the Cantonese lexicon but showed unconventional applications by the ASD speakers. For example, the use of 遺跡 *wai4zik1* “historical remains” in 嗰個睡蓮個遺跡*go2 go3 seoi6lin4 go3 wai4zik1* (“the historical remains of those water lily leaves”) possibly indicated the remaining or broken parts of the water lily leaves shown in the storybook. Such an atypical usage may reflect the semantic weakness of the ASD group in making proper reference to the prototypical lexical items (Eigsti et al., 2007; Volden & Lord, 1991). Additionally, the ASD group produced some metaphors or similes that were less likely to be produced by typical adult speakers. For example, in the storytelling task, an autistic speaker referred to the flying leaves as 太空船*taai3hung1 syun4* “spacecraft”. It was considered slightly unusual because the leaves were flying low over the ground rather than navigating the universe as depicted in the storybook. This might also be possible that the ASD individuals are more creative in generating novel metaphors than the NT controls (Kasirer & Mashal, 2014).

### Group Differences in Grammatical Measures and Mazes

The absence of a significant group difference in morphosyntactic errors may be related to the reduced inflectional morphological system in Chinese. For example, tense errors are prevalent in English-speaking individuals with ASD, while in Chinese tense markers are absent. The functors in Chinese such as aspect markers and sentence final particles are not obligatory. For example, the use of the aspect marker *zo2* and the verb particle *saai3* in the following utterance represent “finished”.

**Table:** 

*ngo5*	*sik6*	*zo2/saai3*	*faan6*	*laa3*
I	eat	ASP/VPRT	rice	SFP
“I finished the meal.”				

But they entailed subtle differences such that *zo2* in the example highlighted on the action has been attempted whereas *saai3* focused on the fact that the action is completed. Both are acceptable and grammatical and the use depends on speaker’s intention and focus. The optional nature makes it hard to confirm that certain production was not correct, resulting in a similar performance in the production of morphosyntactic errors in the two groups. It may be possible that a standardized assessment, in particular a comprehension or a judgement test, may better uncover their grammatical difficulties in controlled conditions. The ASD group, however, demonstrated a significantly shorter MLU, which may reveal that their grammatical abilities are different from the controls. The large within-group variability also indicated that that some autistic individuals’ productions were far from typical performance.

### Relationship of GUL with Grammar and Mazes

GUL was expected to be linked to the grammatical ability given its generative nature. In this study, the P_GUL was positively correlated with MLU in the NT group while the P_GUL increased with P_mazes in the ASD group. And no significant relationship between P_GUL and occurrence of morphosyntactic errors in both groups. The positive correlation between P_GUL (and P_pedantic language) and MLU observed in the NT group suggested that those who used complex vocabulary (i.e., pedantic language) also used more advanced syntax (i.e., longer MLU). As pointed out earlier, pedantic language produced by the controls mainly concentrated in their narratives. It may be possible that more formal vocabulary was used to enrich the narration, representing a form of language sophistication. The observations concerning the ASD group did not align with the pattern in the NT group, nor the argument suggested by Volden and Lord (1991), who identified a positive correlation between GUL and utterance complexity (MLU); and association linked with weaker grammar (Eigsti et al., 2007). The absence of a significant correlation between P_pedantic language and MLU, and between P_pedantic language and P_morphosyntactic errors revealed that the use of pedantic form does not necessarily imply more advanced, or deficient language skills. Instead, the positive correlation between the use of GUL or pedantic language and P_mazes indicates that autistic speakers who produced more pedantic language are more likely to produce more mazes, revealing a different nature of GUL use when compared to the NT group. Luyster et al. (2022) explained that there is a trade-off between accuracy and efficiency during conversation among autistic speakers who tend to pursue accuracy over efficiency. When the speaker aims to select the most suitable vocabulary, there would be more relevant candidates being activated. With the two available informal and formal varieties (colloquial Cantonese and MSC), many candidates may be activated at the same time which in turn tax their cognitive resources in utterance planning, and more mazes may therefore be produced. Production of excessive dysfluencies in ASD has been widely reported and may related to their difficulties in constructing grammatical utterances and coherent speaking turns as well as finding the correct lexical items (Wiklund & Laakso, 2021). In this case, autistic individuals might have to select specific words and sacrificed fluency.

Another possible explanation for the discrepant findings with previous studies may be related to the age of the participants being studied. In previous studies, autistic participants were recruited from a large age range and were still experiencing a period of rapid language growth (Volden & Lord, 1991: 6–18 years old; Eigsti et al., 2007: 39–78 months). The widely distributed performance on GUL and MLU and the developing language skills in their young cohorts may lead to the robust correlation between the two measures.

### Limitations and Future Studies

In the present study, grammatical ability was viewed as a continuous measure and evaluated in a production task. Future studies may focus on tapping participants’ grammatical skills using a standardized assessment with a receptive task. Given the optional nature of the various morphosyntax in Chinese, the large within-group variability in the ASD group may support a subgroup analysis – separating those with and without clinical concern of language difficulties. Further, this study included genres of narration, monologue of picture description, and conversational dialogue. Future studies can focus on conversational speech so as to control for the effect of genre such that the intentional use of sophisticated vocabulary in NT controls can be distinguished from the pedantic use by the ASD speakers. Finally, future research on the types of neologism (e.g., phonological vs. morphological) can be examined to shed light on the word learning patterns of the autistic speakers.

## Conclusions

This study used spontaneous speech samples to investigate GUL produced by Cantonese-speaking adults with and without ASD, and examine whether the generative nature of their GUL reflected their grammatical performance. As predicted, the autistic adults were more likely to produce GUL than the NT controls, generating a larger proportion of neologisms, idiosyncratic phrases, and especially pedantic language. The pattern of GUL subtypes was also partly modulated by Cantonese-specific features that the correspondence between the oral (informal) and written (formal) languages is low. Moreover, the ASD group also exhibited some issues in grammar as evidenced by their shorter MLU, and a trend of more frequent morphosyntactic errors. GUL in the NT group appeared to be a form of sophisticated language use. However, in the ASD group, the positive association of GUL with grammar skills (MLU) previously found in the English literature was not observed (Volden & Lord, 1991), nor a relation between GUL and morphosyntactic difficulty (Eigsti et al., 2007). The use of GUL among the ASD speakers was more related to maze production instead, implying that their GUL production may be a typical autistic feature rather than an indicator of intentional language use of advanced language skills.
